# Impact of Antidepressant Use on Healthcare Utilization among Individuals with Type 2 Diabetes and Depression Symptoms in the United States: Sociodemographic, Clinical, and Behavioral Factors Matter

**DOI:** 10.3390/ijerph15091904

**Published:** 2018-09-01

**Authors:** Ammena Y. Binsaleh, Alexandra Perez, Ioana Popovici, Silvia E. Rabionet

**Affiliations:** 1 College of Pharmacy, Nova Southeastern University, Davie, FL 33328, USA; ab2420@mynsu.nova.edu (A.Y.B.); alperez@nova.edu (A.P.); ip153@nova.edu (I.P.); 2 Princess Nourah Bint Abdulrahman University, Riyadh 11671, Saudi Arabia; 3 School of Public Health, University of Puerto Rico, San Juan, PR 00936, USA

**Keywords:** diabetes and depression comorbidity, antidepressant use, healthcare utilization, type 2 diabetes, depression symptoms, National Health and Nutrition Examination Survey, healthcare disparities

## Abstract

Individuals with diabetes are twice as likely to struggle from depressive symptoms than individuals without diabetes. However, this joint condition is undertreated in nearly two-thirds of patients. Failure to monitor the comorbidity may lead to suboptimal therapy. This study evaluated the association of antidepressant use with healthcare utilization in a national sample of patients with type 2 diabetes and depression symptoms in the United States. It further assessed the differences in sociodemographic, clinical, and behavioral factors between those who use antidepressants and those who do not. This study was a secondary data analysis using the National Health and Nutrition Examination Survey (NHANES) for the period 2005–2014. To assess if there were significant differences in sociodemographic, clinical, and behavioral factors between those who were taking antidepressants or not, Chi Square and independent t-tests were used. To assess if there was a significant association between antidepressant use and healthcare utilization, univariate and multivariate regression analyses were conducted. Of the 955 participants, only 33% were on antidepressants. There were significant differences in sociodemographic, clinical, and behavioral factors among those who used antidepressants and those who did not. Regardless of antidepressant use, the study population had access to health care. Those on antidepressants had fewer diabetes specialists’ visits and more mental health care. There might be underlying health care disparities related to the use of, and access to, antidepressants. Further studies are needed to comprehensively explore the management of these comorbidities.

## 1. Introduction

Individuals with diabetes are twice as likely to struggle from depressive symptoms than individuals without diabetes [1]. In the United States, 21% of patients with type 1 diabetes and 27% of patients with type 2 diabetes suffered from depressive symptoms [2,3]. The joint condition is undertreated and not recognized in nearly two-thirds of patients with both illnesses [4]. There is a bidirectional adverse interaction between diabetes and depression. Physiological, psychological, and behavioral factors play a critical role in the relationship between the two conditions [5].

Diabetes and depression comorbidity may produce negative health outcomes in patients, including poor symptom control, increased loss of work, and disability [5]. In addition, the mortality rate was higher among patients with both conditions. Persons with both diabetes and depression have a risk of early mortality 2.3 times higher than persons with diabetes who do not have depression [3,6].

Patients with depression are treated with antidepressants, psychotherapy, or a combination of both. These are the same treatment options for patients suffering from both depression and type 2 diabetes. Serafini, et al. reported that conditions such as chronic stress and severe depression are associated with structural brain alterations that include loss of dendritic spines and synapses, decreased dendritic arborization, along with diminished glial cells in the hippocampus. Antidepressants medications are generally recommended to counter stress-induced structural alterations amplifying dendritic arborization and synaptogenesis [7]. These medications are also used to regulate neurotransmitters such as serotonin, norepinephrine, and dopamine in the brain [8]. The impact of depression treatment among individuals with type 2 diabetes and depression symptoms in the United States is largely unknown.

Comprehensive diabetes medical evaluation started to incorporate depression screening, thus becoming one of the standards of medical care for diabetes in the US [9]. However, there are no specific pharmacotherapy guidelines or treatment plans recommended for those diagnosed with the comorbidity of diabetes and depression. This study focused on the health care utilization among individuals with both type 2 diabetes and depression, comparing those who are using antidepressants versus those who are not using antidepressants in the US. It explored whether individuals with type 2 diabetes and depression symptoms were receiving care, and if this had an impact on healthcare use. This research will provide baseline knowledge for clinicians and policy makers to take action and implement guidelines for managing patients with type 2 diabetes and depression symptoms. It further assessed the sociodemographic, clinical, and behavioral factors that mediated between the association of antidepressant use and health care utilization. Specifically, this study aimed to answer the following research questions: (i) Are there differences in sociodemographic, clinical, and behavioral factors between those who use antidepressants and those who do not? and (ii) Is there a significant association between antidepressant use and healthcare utilization in patients with type 2 diabetes and depression symptoms? We hypothesized that there was no difference in the specified healthcare utilization outcomes across antidepressant and no antidepressant study groups. 

The results of the study revealed differences in the sociodemographic, clinical, and behavioral factors that exist among patients with type 2 diabetes and depression symptoms, comparing those who use antidepressant and those who do not. Such findings have the potential to assist healthcare professionals in better tailoring their interventions, considering specific factors when prescribing antidepressants to this population and avoid underlining disparities. In addition, the findings of the study showed the healthcare utilization patterns among those who use antidepressants versus those who do not use antidepressants and indicated if they were monitored by healthcare professionals.

## 2. Materials and Methods

### 2.1. Research Design

This study evaluated the association of antidepressant use with healthcare utilization in a nationally representative sample of patients with type 2 diabetes and mild to severe depressive symptoms in the United States population. It was a secondary data analysis using the National Health and Nutrition Examination Survey (NHANES) database using cohorts for the period 2005–2014. NHANES is conducted by the National Center for Health Statistics (NCHS), which is part of the Centers for Disease Control and Prevention (CDC). It is a population-based survey that gathers data on the health and nutrition status of the US household population. Complex, multi-stage sampling design is used to select participants in NHANES. The survey data is collected through home interviews and health examinations. Participants are asked questions about health status, disease history, and diet. The health exams are conducted by a trained medical team in Mobile Exam Centers (MECs) that travel across the nation [10]. Persons who are institutionalized, live in nursing homes or abroad, or are serving in the armed forces are excluded from the sample.

### 2.2. Study Sample and Inclusion Criteria

For the purpose of this study, adults who were aged 20 years or older, had a type 2 diabetes diagnosis, and reported depression symptoms were included. NHANES uses 20 years old as the cut-off for classifying participants as adults. The type 2 diagnosis was established if the participants indicated that a doctor diagnosed them with diabetes when they were 20 years or older. The presence of depression symptoms was established by scoring the participants’ answers to the Patient Health Questionnaire (PHQ-9). If the patients scored less than 5, they were excluded because of the absence of depression symptoms. Patients who scored 5–9 (mild depression symptoms), 10–14 (moderate depression symptoms), 15–19 (moderately severe depression symptoms), and above 20 (severe depression symptoms) were included in the study [11]. A sample of 955 adults with type 2 diabetes and mild to moderate depression symptoms were included in the current study.

### 2.3. Variables and Measurements

Outcome variables. In order to answer the research questions, variables related to healthcare utilization were selected. Each outcome variable captured a different aspect of healthcare utilization. These variables included general access to health care, professional healthcare use, and hospitalization. The variables were selected because they captured different dimensions of healthcare utilization. A summary of the healthcare utilization variables and the operational definitions used is presented in Table 1.

Independent variable. The main independent variable for the study was the use of antidepressants. It was used as a dichotomous variable, 1 for yes, 0 otherwise. Patients were categorized as users if they were taking one or more of the following drug classes: Selective Serotonin Reuptake Inhibitors (SSRIs), Serotonin Norepinephrine Reuptake Inhibitors (SNRIs), Triyclic Antidepressants (TCAs), Monomine Oxidase Inhibitors (MAOIs), atypical antidepressants, and serotonin modulators in the last 30 days prior to survey interview. Data for this variable were extracted from the drug information files in NHANES. It is not possible to verify if the patients take the drug on a regular basis. This variable does not measure the drug schedule or compliance with treatment.

Covariates. The study included three categories of covariates: sociodemographic, clinical, and behavioral factors. The selection of these covariates was based on a comprehensive literature review.

Sociodemographic covariates. Age, gender, race/ethnicity, education level, family Poverty to Income Ratio (PIR), and healthcare insurance were used. All the variables other than healthcare insurance coverage were extracted from the demographic file in the NHANES database. The data on healthcare insurance coverage was obtained from the health insurance questionnaire.

Clinical covariates. The study considered a set of important clinical factors related to the progression of diabetes. The measured covariates were: duration of diabetes, glycemic control, diabetes-treatment intensity, and vascular complications. Comorbidity was explored by including severity of depression symptoms and presence of chronic conditions. Duration of diabetes was calculated by subtracting the age of onset of diabetes from the age at screening; age at screening was obtained from the demographic file and age of onset of diabetes was obtained from the diabetes questionnaire. Glycemic control was extracted from the glycohemoglobin laboratory data. Diabetes-treatment intensity was extracted from the drug information file. Diabetes-related macrovascular complications were obtained from the medical conditions file and microvascular complications were obtained from the albumin and creatinine laboratory data. The severity of depression symptoms was extracted from the mental health depression screener file and the severity of comorbidity was obtained from the medical conditions file.

The study included three behavioral covariates related to weight and exercise to capture the self-care dimension of diabetes. Body mass index was extracted from the body measures examination data. Diet quality was obtained from the diet behavior and nutrition file, and the recreational physical activities were obtained from the physical activity file.

### 2.4. Statistical Analysis

The research questions guided the selection of the statistical analysis. Significant differences in sociodemographic, clinical, and behavioral factors between persons taking antidepressants and persons not taking antidepressants were determined using Chi Square and independent t-tests.

The association between antidepressant use (independent variable) and healthcare utilization (outcome/dependent variable) among patients with type 2 diabetes and depression symptoms were explored using univariate and multivariate linear and logistic regression models. Seven outcome variables were explored under healthcare utilization. The study used the Statistical Package for the Social Sciences (SPSS-IBM version 24, Armonk. NY, USA) to analyze the data. Significance was established at 0.1, due to the exploratory nature of this research. Estimates were calculated by adjusting for complex sampling design and were representative of the United States population.

## 3. Results

Characteristics of patients with type 2 diabetes and depression symptoms will be described at the outset, followed by results corresponding to the research questions. Addressing the first research question, the data presented the differences in sociodemographic, clinical, and behavioral factors between type 2 diabetic patients with depression symptoms who used antidepressants and those who did not use antidepressants. To answer the second question, univariate and multivariate regression analyses were used to assess the association between antidepressant use and health care utilization.

### 3.1. Characterization of Patients with Type 2 Diabetes and Depression Symptoms

This study included 955 adults with type 2 diabetes and depression symptoms. About one-third of them used antidepressants (33%). Over one-half were women (66%), Non-Hispanic Caucasian (59%), had less than a college education (89%), and had health insurance coverage (85%). On average, they were 59 years old and had a family poverty income ratio of above 2. Refer to Table 2.

On average, patients with type 2 diabetes and depression symptoms suffered diabetes for over nine years and, were primarily on oral antidiabetics (56%), insulins (10%), both (11%), or were taking none of these medications (24%) (the percentages exceed 100% due to rounding). They were more likely to have macrovascular complications (29%) than advanced microvascular complications (4%), and scored 4 on average in severity of comorbidity. Slightly more than one-half of these patients had controlled diabetes (54%). Among the sample population, about 60%, 24%, 11% and 4% had mild, moderate, moderate severe, and severe depression symptoms, respectively.

The average body mass index for the entire sample was 34.73 kg/m^2^ and more than one-half were following a healthy diet (56%). Nearly one-third were participating in moderate to vigorous physical recreational activities (29%). 

On average, patients in the sample received healthcare approximately five (Standard Deviation (SD) = 3.58) times in the past year. About 77% of patients visited a physician or specialist to manage their diabetes; they visited this physician or specialist an average of 4 times (SD = 9.76) in the past year. About 36% of the patients had visited a diabetes specialist that included diabetes nurse educators or dieticians in the past year. Only 12.9% of the study population had visited a mental health professional during the same time period. Finally, 29% of all participants had been hospitalized. Those admitted to a hospital were hospitalized, on average, 0.55 (SD = 1.06) times in the past year.

### 3.2. Sociodemographic, Clinical, and Behavioral Differences among Antidepressants Users and Non-Users

Sociodemographic and antidepressant use. There were significant differences in gender, race/ethnicity, and health insurance coverage between patients who were and were not on antidepressants (χ^2^ test, *p* < 0.01), as illustrated in Table 2. Compared to patients who were not on antidepressants, those on medication had a greater representation of women (75.7% versus 61.3%), non-Hispanic Caucasian (71.4% versus 52.3%), and had more health insurance coverage (91.2% versus 81.9%). No significant differences were detected for age (independent t-test, *p* > 0.1), family poverty income ratios (independent t-test, *p* > 0.1), or educational level (χ^2^ test, *p* > 0.1) between those who used antidepressants and those who did not use them (Refer to Table 2).

Clinical factors and antidepressant use. Within the two groups, there were significant differences in vascular complications (χ^2^ test, *p* < 0.05) and severity of depression symptoms (χ^2^ test, *p* < 0.01). Macrovascular complications were more likely to occur within those who were on antidepressants (32.7% versus 27.1%), but microvascular complications were more common in patients who were not on antidepressants (2% versus 5.2%).

Most individuals with mild depression symptoms were not using antidepressants (67%). Only 32.2% of individuals with moderate depression symptoms were using antidepressants and 15% of those with moderately severe depression used antidepressants. About 5.7% of patients with severe depression were using antidepressants (Refer to Table 3). No significant differences were found in duration of diabetes (Independent *t*-test, *p* > 0.1), diabetes-treatment intensity (χ^2^ test, *p* > 0.1), glycemic control (χ^2^ test, *p* > 0.1), or severity of comorbidity (Independent t-test, *p* > 0.1) among the two groups (Refer to Table 3).

Behavioral factors and antidepressant Use. Differences in behavioral factors among type 2 diabetic patients with depression symptoms who used antidepressants and those who did not use them were summarized in this section (Refer to Table 4). When patients taking and not taking antidepressants were compared, significant differences emerged in body mass index (Independent t-test, *p* < 0.01), diet quality (χ^2^ test, *p* < 0.1), and physical recreational activities (χ^2^ test, *p* < 0.01). Average body mass index among patients using antidepressants was higher than that for those did not use them (35.8 kg/m^2^ versus 33.5 kg/m^2^). Patients using antidepressants were also less likely to follow a healthy diet (50.5% versus 58.7%) and less likely to engage in moderate to vigorous physical recreational activities (19.5% versus 34.3%).

### 3.3. Association between Antidepressant Use and Healthcare Utilization

General access to healthcare. Univariate linear regression analyses showed that those who were on antidepressants experienced a significant increase of 0.893 in the number of times they received healthcare in the past year compared to those who were not on antidepressants in this population (*p* < 0.05). However, after controlling for sociodemographic, clinical, and behavioral factors, there was no significant association between use of antidepressants and the average number of times receiving health care in past year (Multivariate linear regression analysis, *p* > 0.1) (Refer to Table 5).

Professional healthcare use. The evidence also revealed no significant associations between antidepressant use and either physician visits to manage diabetes in past year (Univariate logistic regression analysis, *p* > 0.1) or number of physician visits in past year (Univariate linear regression analysis, *p* > 0.1) among patients with type 2 diabetes and depression symptoms. The associations continued to lack significance after controlling for all covariates (Multivariate logistic regression analysis and multivariate linear regression analysis, *p* > 0.1).

The association between antidepressant use and visiting a diabetes specialist (diabetes nurse educator or dietitian) in the past year was significant (Univariate logistic regression, *p* ≤ 0.05, (Odds Ratio (OR) = 0.68)); patients on antidepressants were 32% less likely to visit a diabetes specialist than who were not on antidepressants. After controlling for sociodemographic, clinical, and behavioral factors, the association remained significant with a value of 36.7% (Multivariate logistic regression, *p* ≤ 0.05, OR = 0.633) (see Table 6).

There association between antidepressant use and visits to a mental health professional among the study population was significant. Patients on antidepressants were 3.5 times more likely to visit a mental health professional in the past year than patients who were not on antidepressants (Univariate logistic regression, *p* < 0.01, OR = 3.5). After controlling for sociodemographic, clinical, and behavioral factors, the association continued to be significant and became stronger; patients taking antidepressants were 4.8 times more likely to visit a mental health professional (Multivariate logistic regression, *p* < 0.01, OR = 4.8) (Refer to Table 6).

Hospitalization. Among patients with type 2 diabetes and depression symptoms, there was no significant association between antidepressant use and overnight hospitalizations (Univariate and multivariate logistic regression, *p* > 0.1) or number of hospitalizations (Univariate and multivariate linear regression, *p* > 0.1) (Refer to Table 7).

## 4. Discussion

This study focused on adults with comorbidity of type 2 diabetes and depression symptoms. These patients were at risk of complications, requiring proper treatment and follow up. Although both diabetes and depression clinical guidelines have highlighted the need for routine screening for depression and indicated special treatment consideration for adults with diabetes, there are no specific pharmacotherapeutic recommendations provided for this population. Therefore, the current results provide information that may be used by policy makers and healthcare professionals to devise treatment guidelines and standards of care for adults with type 2 diabetes and depression comorbidity.

### 4.1. Differences in Sociodemographic, Clinical, and Behavioral Factors within Antidepressant Users and Non-Users

Of the 955 participants, only one-third were on antidepressants. This highlighted the underutilization of pharmacological-based treatment in this population, even when this is considered the treatment of choice for patients with mild to moderate depression and a must for patients with severe depression [12]. Sociodemographic, clinical, and behavioral factors among antidepressant users and non-users partly explained the underuse of the pharmacological treatments.

Sociodemographic characteristics and antidepressant use. Examining sociodemographic factors revealed differences that existed in patients with type 2 diabetes and depression symptoms. Out of the six sociodemographic factors that were investigated, three were different across treatment groups: gender, race, and healthcare insurance. Women were more likely to use antidepressants. A study conducted among individuals with depression in the United States showed similar results. The researchers reported that a higher proportion of females were using antidepressants, specifically selective serotonin reuptake inhibitors [13]. However, other studies have shown different results. In Spain, researchers found no significant differences in gender among patients with type 2 diabetes and depression symptoms who used citalopram and those who did not use an antidepressant [14].

Non-Hispanic Caucasians were more likely than other ethnic groups to use antidepressants. The proportion of Hispanics and non-Hispanic Blacks on antidepressant therapy was significantly lower than that of non-Hispanic Caucasians. A previous study showed similar results among patients with type 2 diabetes and depression symptoms. That study found that Mexican Americans and Blacks were significantly less likely to use antidepressants than Whites. That study was the first pharmacological study to examine antidepressant use in racial/ethnic groups with type 2 diabetes and depression symptoms in the US [15]. An earlier study conducted among individuals with depression reported that Blacks were less likely to use antidepressant than Whites [16]. The findings of the current study underlined the need to further explore underlying disparities within minority groups in seeking health care and treatment. Future research should incorporate medical confirmation of depression and prescription of antidepressants and not solely rely on self-reported data.

The results also showed that the proportion of health insurance coverage was higher among patients who were on antidepressants. A previous study had shown that the use of prescription medication tended to increase with greater insurance coverage [17].

The evidence here showed no significant differences in age, family poverty income ratios, and educational level between the two groups; in an earlier study, Brown et al. (1995) had found that advanced age, income, and educational level were not associated with antidepressant use [16].

Clinical factors and antidepressant use. Of the six clinical factors investigated, there were two that were different among those who used antidepressants compared to those who did not use antidepressants. The prevalence of microvascular complications was lower in patients who took antidepressants. There was also a significant difference in the severity of depression symptoms between patients who took and did not take antidepressants. Results showed that, among those who were not using antidepressants, two-thirds had mild depression and the rest moderate to severe depression symptoms. This study group may not have had a depression diagnosis or been prescribed pharmacological treatment yet. In contrast, in the antidepressant treatment group less than one-half had mild depression and the rest moderate to severe depression symptoms. It could be assumed that the latter group had a depression diagnosis and they were failing pharmacological treatment. This highlighted the need for optimization of therapy.

There were no significant differences between the two groups in duration of diabetes, diabetes-treatment intensity, diabetes control (glycohemoglobin level), or severity of comorbidity. These findings were consistent with a study conducted by Nicolau et al. (2013) in Spain that showed there was no significant differences in duration of diabetes, diabetes treatment (insulin and oral agents), glycohemoglobin level among type 2 diabetic patients with depression symptoms, who used an antidepressant (citalopram) and those who did not use an antidepressant [14].

Behavioral factors and antidepressant use. Significant differences in body mass index, diet, and physical recreational activities were found between antidepressant users and non-users. The results showed that patients with type 2 diabetes and depression symptoms who were using antidepressants had a greater body mass index (35.8 kg/m^2^) than those who were not using antidepressant. As in a previous study, there was a significant difference in the body mass index among those who used an antidepressant (citalopram) compared to those who did not use an antidepressant [14].

Weight gain has been reported as a side effect of antidepressants use [12]. In particular, SSRI, TCAs, and MAOI antidepressants cause weight gain and thus resulted a higher body mass index. Health care providers should discuss with patients taking antidepressants the necessary approaches to achieve and maintain weight control. Treatment guidelines recommend that patients on antidepressants need to both exercise regularly and follow a healthy diet (following dietitians’ recommendations). Patients with depression symptoms require longitudinal weight monitoring that requires collaboration between primary care providers and dietitians [12]. It is, therefore, critical to consider the side effects of different classes of antidepressants when prescribing this option to patients with type 2 diabetes and depression symptoms; they must be apprised that the medication might increase weight and worsen their health outcomes.

This is especially important in light of the finding that patients using antidepressant were less likely to follow a healthy diet or engage in moderate or vigorous physical recreational activities than patients not taking antidepressants. This confirmed a previously reported negative relationship between depression and both physical activity and diet quality [18,19]. The negative relationship persisted even after taking antidepressants. The relationship between physical activity and depression treatment must be further explored in future research.

### 4.2. Association between Healthcare Utilization and Antidepressant Use

The findings of this current study showed that, whether or not they were taking antidepressants, patients with type 2 diabetes and depression symptoms had access to and used the healthcare system. On average, they reported receiving healthcare five times in the past year. Three quarters of patients visited a general practitioner or an endocrinologist (specialist), on average, four times to manage diabetes. Most patients were receiving diabetes care whether or not they were taking antidepressants. One previous study showed there was significant association between number of doctor visits and antidepressant use. Brown et al. (1995) found that, after controlling for differences in gender, race, and self-perceived health, there was a significant association between use of antidepressants and number of doctor visits among the elderly with depression. This finding, which was not consistent with the results of the current study, could be explained by variations over time and differences in the population studied. Brown et al. conducted their study more than 20 years ago. Moreover, they focused only on the elderly with depression, so their findings might not apply to estimates for the population of theUnited States as a whole [16].

Taking advantage of the high rates of physician visits, patients with type 2 can be screened for depression. Physicians could prescribe the necessary medications, including antidiabetic and antidepressant medications; follow up on the patients’ progress; and prevent the magnitude of both diabetes and depression complications that might appear if these patients were not properly monitored.

Patients suffering from diabetes benefit from the complementary professional health education and care provided by non-physician specialists. Yet in this study, patients who were on antidepressants were 36.4% less likely to visit diabetes nurse educators or dieticians than those who were not on antidepressants. This finding underscored the need for encouraging visits to diabetes nurse educators or dieticians capable of providing advice on self-care and healthy diets to complement the joint antidepressants and antidiabetic therapy.

It is not surprising to learn from the results of this study, that patients with type 2 diabetes and depression symptoms who were on antidepressant were more likely to be monitored by a mental health professional than those who were not on antidepressants. However, regardless of medication use, 87% of these patients were not seeing a mental health professional and 77% were not taking antidepressants. Furthermore, this may be a consequence of the mental health services shortage in the United States [20] and of the fragmented nature of the health care system [21]. Stigma or shame is probably an important barrier hindering patients from being monitored by a mental health professional. This finding supports the need to expand the number of and access to physicians of different specialties such as psychiatrists and promote practice collaboration among these physicians. Mental health professionals should be integrated into these teams.

Regardless of whether patients were on antidepressants or not, nearly to one third of the population (29%) were hospitalized in the past year. Individuals with the comorbidity of type 2 diabetes and depression were susceptible to complications. If not monitored, such complications could lead to costly hospitalizations.

### 4.3. Limitations

There were several limitations in the study. Its results might not be generalized beyond the period covered, namely, 2005–2014 for the United States population. The study followed a cross-sectional design, so establishing causality between the variables was not appropriate. NHANES database, used as the source, is based on self-reported data subject to recall bias. Stigma might have caused respondents to hide their true symptoms of depression in the Patient Health Questionnaire-9, and this might have altered the results. Although participants who reported mild, moderate, moderately severe, and severe depression symptoms were included, the database did not allow for verification of the depression diagnosis by a health professional. Reasons for the difference in depression symptom distributions among antidepressant users and non-users must be noted. Moderate to severe depression symptoms were more prevalent among those being treated. Although these patients may already have a depression diagnosis their treatment is either failing or has not reached onset of action. In contrast, those who were untreated were much more likely to have mild depression symptoms so they are patients who either may not have had a diagnosis of depression yet or had a diagnosis in the past but discontinued treatment. Therefore, caution is warranted when extrapolating this study’s conclusions to a population with a diagnosis of both type 2 diabetes and depression. This study was meant to evaluate the effect of being on antidepressant treatment, not the effect of antidepressant drug classes or their treatment duration. The selection of covariates was guided by the literature. However, their definition and measurement were constrained by the criteria and parameters used in the survey methodology. Results might have differed if other sets of covariates had been considered.

## 5. Conclusions

This study found some important issues and considerations that may have implications for care and policy, in terms of positively affecting patients suffering from diabetes and depression symptoms. First, it is important to develop specific therapeutic guidelines for patients with the comorbidity of diabetes and depression. An important step in the right direction may be acknowledging the bi-directionality of the two conditions, i.e., the effects of diabetes on depression and vice versa, in adopting guidelines for these comorbidities.

Most patients suffering from the two conditions had health insurance, and those using antidepressants were more likely to have insurance. This coverage increases the feasibility of launching educational campaigns to encourage patients to visit providers. Health care navigation programs can also benefit this population. Attention to service coordination and collaboration might also highly benefit this population. As changes are made to the Affordable Care Act, it is important that practitioners, policy makers and patient advocacy groups weigh the benefits of having coverage in order to enhance and ensure access to care.

Patients with depression should be reexamined every three to four months to ensure receiving appropriate therapy. Psychiatrists should conduct comprehensive assessment of general medical conditions to evaluate factors aggravating depressive symptoms and collaborate with other healthcare professionals. Timely and communication would enhance overall treatment, tackle medical conditions, and provide vigilance against side effects and harmful risks [12].

In summary, there were significant differences in terms of sociodemographic (gender, race, and health insurance coverage), clinical (diabetes related complications and severity of depression), and behavioral factors (body mass index, diet, and recreational physical activities) among those who used antidepressant and those who did not use antidepressants within the population with type 2 diabetes and depression symptoms.

Regardless of antidepressant use, the study population had access to the healthcare system, most were visiting physicians to manage diabetes and were receiving diabetes care, and nearly one-third of the population had been hospitalized in the past year. Patients who used antidepressants had fewer diabetes nurse educators and dietitians’ visits. These professionals are important to promote self-care behavior accompanied by the necessary pharmacological treatment. Patients who used antidepressants were more likely to be monitored by a mental health professional. However, most of this population made fewer mental professional visits, a finding that could be related to stigma and shame-related barriers and the underutilization of antidepressants medications.

The results indicated that there might be underlying health care disparities related to the use of, and access to, antidepressants. Shortage of mental health providers may be contributing to this gap in care and must be addressed at a national level. There is a need for developing treatment guidelines and medical care standard that emphasize collaboration among different specialties and level of care when serving patients with diabetes type 2 and depression comorbidity. Further studies are needed to systematically and comprehensively explore the management of the disease and the symptoms when the comorbidities are present.

## Figures and Tables

**Table 1:** Healthcare Utilization (Outcome Variables) and its Measurements among Individuals with Type 2 Diabetes and Depression Symptoms.

Outcome Variables	Operational Definition
General Access to Healthcare	
Number of times received healthcare	Number of times that the person visited a healthcare professional at a doctor’s office, clinic, or some other place in the past 12 months
Professional Healthcare Use	
Physician visits	Visited a general or specialist physician to manage diabetes in the past 12 months (yes/no).
Number of physician visits	Number of times that they visited a general or specialist physician in the past 12 months.
Diabetes specialist visit	Visited a diabetes specialist, in the past 12 months. Included diabetes nurse educators or dieticians that provided diabetes self-care knowledge and weight management skills through diet and physical activity (yes/no). Physicians and specialists were not included in this measurement.
Mental health professional visit	Visited a mental health professional including a psychologist, psychiatrist, psychiatric nurse, or clinical social worker in the past year (yes/no).
Hospitalization	
Overnight hospitalization	Had an overnight admission in the past 12 months (yes/no).
Number of overnight hospitalizations	Number of overnight admissions in the past 12 months.

**Table 2:** Sociodemographic Characteristics of Patients with Type 2 Diabetes and Depression Symptoms.

Variable	Total (*n* = 955)	Taking Antidepressants (*n* = 278)	Not Taking Antidepressants (*n* = 677)
Age (years)	58.69 (12.58)	58.84 (12)	58.54 (12.82)
Gender *			
Women (%)	66.1	75.7	61.3
Men (%)	33.9	24.3	38.7
Race *			
Hispanics (%)	17.1	11.2	20
Caucasians (%)	58.6	71.4	52.3
Black (%)	17.8	12.7	20.3
Other race (%)	6.5	4.7	7.4
Family poverty income ratios	2.15 (1.32)	2.10 (1.26)	2.21 (1.35)
Education			
College or above (%)	10.8	10.6	10.9
Less than college (%)	89.2	89.4	89.1
Healthcare Insurance *			
Covered by health insurance (%)	85	91.2	81.9
Not covered by insurance (%)	15	8.8	18.1

Notes: Chi square tests were used for categorical variables, Independent t-tests were used for continuous variables. Standard deviations were reported in parentheses for continuous variables. * Statistically significant, *p* ≤ 0.01.

**Table 3:** Clinical Characteristics of Patients with Type 2 Diabetes and Depression Symptoms.

Variable	Total (*n* = 955)	Taking Antidepressants (*n* = 278)	Not Taking Antidepressants (*n* = 677)
Duration of diabetes (years)	9.39 (9.33)	9.15 (9.19)	9.51 (9.39)
Diabetes Treatment Intensity			
Insulin only (%)	9.5	11.5	8.6
Oral antidiabetic(s) only (%)	55.7	54.9	56.1
Both (%)	10.5	11.4	10
None (%)	24.3	22.2	25.3
Vascular Complications			
Macrovascular complications (%)	28.9	32.7	27.1
Microvascular complications (%) *	4.2	2	5.2
Glycemic control			
Controlled diabetes (%)	54.7	58.1	53.1
Uncontrolled Diabetes (%)	45.3	41.9	46.9
Severity of Depression Symptoms **			
Mild depression symptoms ^a^ (%)	60.6	47.1	67.2
Moderate depression symptoms ^b^ (%)	23.9	32.2	19.7
Moderate severe depression symptoms ^c^ (%)	11.2	15	9.3
Severe depression symptoms ^d^ (%)	4.4	5.7	3.8
Severity of comorbidity ^e^	3.88 (1.83)	4 (1.83)	3.76 (1.80)

Notes: ^a^ PHQ-9 scores 5–9, ^b^ PHQ-9 scores 10–14, ^c^ PHQ-9 scores 15–19, ^d^ PHQ-9 scores ≥ 20, ^e^ Charlson Comorbidity Index scores (CCI) ranged from 0–32, Mild CCI scores 1–2, Moderate CCI scores 3–4, Severe CCI scores ≥5. Chi square tests were used for categorical variables. Independent t-tests were used for continuous variables. Standard deviations were reported in parentheses for continuous variables. * Statistically significant, *p* ≤ 0.05, ** Statistically significant, *p* ≤ 0.01.

**Table 4:** Behavioral characteristics of patients with type 2 diabetes and depression symptoms.

Variable	Total (*n* = 955)	Taking Antidepressants (*n* = 278)	Not Taking Antidepressants (*n* = 677)
Body Mass Index (kg/m^2^) **	34.73 (8.56)	35.81 (9.31)	33.53 (8.15)
Diet Quality *			
Had healthy diet (%)	56	50.5	58.7
Had unhealthy diet (%)	44	49.5	41.3
Physical recreational activities **			
Engaged in moderate to vigorous recreational activities (%)	29.4	19.5	34.3
Not engaged in moderate to vigorous recreational activities (%)	70.6	80.5	65.7

Notes: Chi square tests were used for categorical variables. Independent t-tests were used for continuous variables. Standard deviations were reported in parentheses for continuous variables. * Statistically significant, *p* ≤ 0.1, ** Statistically significant, *p* ≤ 0.01.

**Table 5:** Coefficients and levels of significance for the association between use of antidepressants and general access to healthcare.

	Number of Times Received Healthcare in Past Year
On Antidepressants	
Uncontrolled effect	*n* = 953 0.893 * (0.025)
Controlling for sociodemographic, clinical, and behavioral factors	*n* = 817 0.101 (0.806)

Note: Numbers without parenthesis were the coefficients of regression analyses. Numbers in parentheses represented the *p*-value. * Statistically significant, *p* ≤ 0.05.

**Table 6:** Coefficients and levels of significance for the association between use of antidepressants and professional healthcare use.

	Physician Visit to Manage Diabetes in Past Year	Number of Physician Visits in Past Year	Diabetes Specialist (Non-Physician) Visit in the Past Year	Mental Health Professional Visit in Past Year
On Antidepressants				
Uncontrolled effect	*n* = 955 OR = 1.09 CI = [0.714–1.665] (0.686)	*n* = 954 0.494 (0.337)	*n* = 950 OR = 0.680 ** CI = [0.463–0.999] (0.050)	*n* = 955 OR = 3.509 *** CI = [2.258–5.453] (<0.005)
Controlling for sociodemographic, clinical, and behavioral factors	*n* = 819 OR = 0.829 CI = [0.497–1.435] (0.471)	*n* = 818 −0.014 (0.98)	*n* = 816 OR = 0.636 * CI = [0.401–0.999] (0.056)	*n* = 819 OR = 4.884 *** CI = [2.537–9.412] (<0.005)

Note: OR, odds ratio. CI, confidence interval. Numbers without parenthesis were the coefficients of regression analysis. Numbers in parenthesis represented the *p*-value. * Statistically significant, *p* ≤ 0.1, ** Statistically significant, *p* ≤ 0.05, *** Statistically significant, *p* ≤ 0.01.

**Table 7:** Coefficients and levels of significance for the association between use of antidepressants and hospitalizations.

	Reported to Have an Overnight Hospitalization in Past Year	Number of Overnight Hospitalizations in Past Year
On Antidepressants		
Uncontrolled effect	(*n* = 955) OR = 1.146 CI = [0.691–1.902] (0.594)	(*n* = 955) 0.152 (0.201)
Controlling for sociodemographic, clinical, and behavioral factors	(*n* = 819) OR = 0.838 CI = [0.468–1.49] (0.546)	(*n* = 819) 0.022 (0.822)

Note: OR, odds ratio. CI, confidence interval. Numbers without parenthesis were the coefficients of regression analysis. Numbers in parenthesis represented the *p*-value.
